# Validation of a menstrual pictogram and a daily bleeding diary for assessment of uterine fibroid treatment efficacy in clinical studies

**DOI:** 10.1186/s41687-020-00263-0

**Published:** 2020-11-13

**Authors:** Claudia Haberland, Anna Filonenko, Christian Seitz, Matthias Börner, Christoph Gerlinger, Helen Doll, Dorothea Wessiepe

**Affiliations:** 1 Market Access, Public Affairs & Sustainability, TA Pulmonology / Innovative WHC, Bayer AG, Building S157, 3.315, 13342 Berlin, Germany; 2 Market Access, Public Affairs & Sustainability, TA Pulmonology / Innovative WHC, Bayer AG, Building S157, R318, 13342 Berlin, Germany; 3 Research & Development, Pharmaceuticals, CD Gynecological Therapies, Bayer AG, Building P300, A422, 13342 Berlin, Germany; 4 Statistical Analytics, Bayer AG, Building P300, A138, 13342 Berlin, Germany; 5 Statistics and Data Insights, Bayer AG, Building P300, A121, 13342 Berlin, Germany; 6 Department of Gynecology, Obstetrics and Reproductive Medicine, University Medical School of Saarland, Homburg, Saar Germany; 7 ICON Patient Centered Outcomes, London, UK; 8 Strategic Lead, Quantitative Science, Clinical Outcomes Solutions, Folkestone, UK; 9 Metronomia Clinical Research GmbH, Muenchen, Germany

**Keywords:** Uterine fibroids (UF), Menstrual pictogram (MP), Uterine fibroid daily bleeding diary (UF-DBD), Alkaline hematin method (AH), Patient-reported outcome (PRO) instruments, Women’s health

## Abstract

**Background:**

To evaluate the psychometric and measurement properties of two patient-reported outcome instruments, the menstrual pictogram superabsorbent polymer-containing version 3 (MP SAP-c v3) and Uterine Fibroid Daily Bleeding Diary (UF-DBD).

Test-retest reliability, criterion, construct validity, responsiveness, missingness and comparability of the MP SAP-c v3 and UF-DBD versus the alkaline hematin (AH) method and a patient global impression of severity (PGI-S) were analyzed in post hoc trial analyses.

**Results:**

Analyses were based on data from up to 756 patients. The full range of MP SAP-c v3 and UF-DBD response options were used, with score distributions reflecting the cyclic character of the disease. Test-retest reliability of MP SAP-c v3 and UF-DBD scores was supported by acceptable intraclass correlation coefficients when stability was defined by the AH method and Patient Global Impression of Severity (PGI-S) scores (0.80–0.96 and 0.42–0.94, respectively). MP SAP-c v3 and UF-DBD scores demonstrated strong and moderate-to-strong correlations with menstrual blood loss assessed by the AH method. Scores increased in monotonic fashion, with greater disease severities, defined by the AH method and PGI-S scores; differences between groups were mostly statistically significant (*P* < 0.05). MP SAP-c v3 and UF-DBD were sensitive to changes in disease severity, defined by the AH method and PGI-S. MP SAP-c v3 and UF-DBD showed a lower frequency of missing patient data versus the AH method, and good agreement with the AH method.

**Conclusions:**

This evidence supports the use of the MP SAP-c v3 and UF-DBD to assess clinical efficacy endpoints in UF phase III studies replacing the AH method.

## Background

Uterine fibroids (UF) are commonly occurring benign tumors of the uterus that originate from smooth muscle cells of the myometrium [1]. The reported prevalence of UF varies from 4.5% to 68.6% across studies [2], thereby making it difficult to determine the true global prevalence of UF.

The majority of women with UF are asymptomatic and may be undiagnosed [1, 3]. Heavy menstrual bleeding (HMB) is commonly reported and, for some women, may lead to UF-related anemia [4–8]. HMB can be severe, has a considerable physical and emotional impact, and can limit women’s participation in professional, domestic, and social activities [9, 10].

In current clinical practice, a woman’s self-perception of her menstrual blood loss (MBL) and the impact of HMB on her health related quality of life (HRQoL) is used to guide the diagnostic and treatment process [11]. A number of daily bleeding diaries (DBDs) are available for use in a variety of gynecological conditions for the woman to rate the extent of blood loss (e.g., from ‘none’ to ‘severe’) but these do not allow assessment of the amount of blood lost. Self-assessment of MBL may not be accurate; quantitative evaluation of MBL in addition to perceived MBL by women may contribute to better clinical care and informed decision-making [12–14].

The alkaline hematin (AH) method is the established method for quantitative assessment of MBL and has traditionally been used to diagnose HMB within clinical trials in agreement with the US Food and Drug Administration (FDA); other regulatory authorities allow pictorial methods [13], which correlate the visual appearance of total menstrual fluid loss on standardized used sanitary products to an estimated MBL volume [15]. The AH method requires women to collect, date, store and send used sanitary products for laboratory analysis of actual blood volume loss (in mL) in a process that can be unfeasible and inconvenient for women, is expensive for laboratory testing and might also be a reason for patients’ non-adherence to the study protocol [15, 16]. Due to such practical limitations, its use has not extended to clinical practice [13]; and in clinical trials, it may be a source for patient non-compliance.

Given the above factors, it follows that there is a need for a semi-quantitative method of MBL assessment, which could serve as an accurate assessment of HMB in routine practice and a convenient, accurate tool in clinical trials. Pictorial blood loss assessment charts (PBAC) s are simple semi-quantitative methods to determine MBL volume. Different PBACs have been developed and have shown variable sensitivity and specificity for detection of HMB, in comparison with the AH method [13, 17, 18]. The menstrual pictogram superabsorbent polymer-containing version 3 (MP SAP-c-v3) (hereafter referred to as the MP), is a PBAC that has been developed for the use with a range of modern sanitary products [15]. It allows the user to assess the visual appearance of blood-stained sanitary protection, using pictograms to provide an estimation of MBL [12]. The MP has been developed for use with the most widely used modern sanitary towels in the United Kingdom and United States [15] that contain superabsorbent polymer (SAP) granules, which can absorb fluid many times their own weight [12]. In a study assessing the validity of the MP, a sensitivity of 82% and a specificity of 92% was found for a diagnosis of HMB determined using the AH [15]. The Uterine Fibroid Daily Bleeding Diary (UF-DBD) has also been developed allowing a subjective assessment of bleeding severity in support of evaluation of treatment efficacy as it enables the subject’s self-assessment of bleeding events that cannot be captured by the AH or the MP methods (i.e., spotting, or any blood lost that is not collected on a sanitary product, and the perceived severity of bleeding). The MP method is nevertheless proposed as the semi-quantitative alternative to the AH method.

Vilaprisan is a novel medical treatment for UF that has been investigated in two phase II studies, ASTEROID 1 and 2. The efficacy of vilaprisan in improving HMB was evaluated via three bleeding assessment instruments: the AH method (ASTEROID 1), MP (ASTEROID 1 and 2), and UF-DBD (ASTEROID 1 and 2). This analysis aims to evaluate the psychometric and measurement properties of the MP and the UF-DBD using data from ASTEROID 1 and ASTEROID 2. Additional analyses include those of missingness and comparability of methods (MP and UF-DBD vs AH, respectively), using data from ASTEROID 1.

## Methods

### Trial designs

ASTEROID 1 (NCT02131662) and ASTEROID 2 (NCT02465814) were randomized, parallel-group, double-blind multicenter studies; full details of the study designs and results of the primary endpoints have been published elsewhere [19, 20]. Patient inclusion and exclusion criteria were the same for both ASTEROID 1 and 2. Women were eligible if aged 18–50 years, with UFs identified by transvaginal or abdominal ultrasound at screening with at least one UF with largest diameter ≥ 3.0 cm and HMB > 80 mL documented by MP during the bleeding episode following the screening visit. Women were excluded if they had one/multiple UF(s) with a diameter exceeding 10.0 cm. For the analyses presented here, all women with available data from the ASTEROID 1 and 2 studies were eligible, regardless of screening failure (for example < 80 mL MBL), drop-out or protocol deviation.

The clinical studies met all local legal and regulatory requirements and were conducted in accordance with the ethical principles that have their origin in the Declaration of Helsinki and the International Council for Harmonization (ICH) guideline E6: Good Clinical Practice (GCP). Analyses of psychometric and other measurement properties were conducted in line with scientific standards including the FDA PRO Guidance for Industry, 2009 [21].

In general, data for the analyses presented here were collected from screening and treatment periods. Objective data were collected using the biochemical AH method, which was the reference measure in blood volume analyses. Patient-reported outcomes (PROs) were collected via the MP, UF-DBD, Uterine Fibroid Daily Symptom Diary version 3 (UF-DSD v3), Uterine Fibroid Impact Scale version 3 (UF-IS v3), Uterine Fibroid Symptom and Quality of Life Questionnaire (UFS-QoL), Short-Form 36 Health Survey Version 2® (SF-36 v2®), and Patient Global Impression of Severity (PGI-S) (Table 1). The PGI-S asks the patient to rate the severity of her UF symptoms on a six-point Likert scale (from “None” to “Very severe”). The PGI-S used in Asteroid 1 and 2 has no recall period and was administered during the Asteroid 1 and 2 study visits.

**Table 1:** Methods used to investigate psychometric and other properties of the MP and UF-DBD

Statistical analysis	PRO scores	Time points	Reference measures	Population	Interpretation
**Item performance/variability – extent to which potential available response option for each item is selected by patients**					
Descriptive statistics	• MP • UF-DBD item level^a^ • Monthly and bleeding episode sum scores	• RND • EOT	NA	All patients, by study	• Distributional properties • Floor and ceiling effects
**Reliability/Test-retest reliability – ability to give reproducible, consistent scores over a short time period in stable patients**					
1. Descriptive statistics, Wilcox signed rank test 2. Intraclass correlation coefficient	• MP • UF-DBD • Monthly and bleeding episode sum scores	• SCR2, RND • T2, EOT	• AH^b^ • PGI-S	Stable patients by study: • AH method: MP and UF-DBD in ASTEROID 1^c^ • PGI-S scores: UF-DBD and MP in ASTEROID 1 and 2	**UF-DBD** • ICC ≥0.50: moderate **MP** • ICC < 0.40: poor • 0.40 to 0.59: moderate • 0.60 to 0.74: good • 0.75+: excellent
**Construct validity – extent to which a scale measures the intended construct**					
***Known-groups validity – ability of measure to discriminate between patient groups differing in levels of condition severity***					
1. Jonckheere-Terpstra test 2. Kruskal-Wallis test	• MP • UF-DBD • Monthly and bleeding episode sum scores	• RND • EOT	• AH^b^ • PGI-S	All patients, by study^c^	Significance of ordered difference between known groups
***Convergent and divergent validity – extent of association between a measure and other measures or variables based on an expected relationship***					
1. Spearman rank correlation 2. Scatterplots	• MP • UF-DBD • Monthly and bleeding episode sum scores	• RND • EOT • pooled RND and EOT	• MP • UF-DBD • UF-DSD v3 • UF-IS v3 • UFS-QoL • SF-36 v2®	All patients, by study	**Strength of correlations** • 0.10 to 0.29: weak • 0.30 to 0.49: moderate • 0.50 to 1.0: strong
**Criterion validity – extent of relation between PRO instrument scores and a known gold standard measure of the same concept**					
1. Spearman rank correlation 2. Scatterplots	• MP • UF-DBD • Monthly and bleeding episode sum scores	• RND • EOT	• AH^b^	All patients with AH measurements	**Strength of correlations** • 0.10 to 0.29: weak • 0.30 to 0.49: moderate • 0.50 to 1.0: strong
**Responsiveness – ability to detect change when a change in the measured concept has occurred**					
1. Spearman rank correlation, Scatterplots 2. Kruskal-Wallis test, Jonckheere-Terpstra test	• MP • UF-DBD • Monthly and bleeding episode sum scores	• RND • EOT	**Correlation analysis** • MP • UF-DBD • UF-DSD v3^d^ • UF-IS v3^d^ • UFS-QoL^d^ • AH^b^ • PGI-S **Definition of change** • AH^b^ • PGI-S	All patients, by study^c^	**Strength of correlations** • 0.10 to 0.29: weak • 0.30 to 0.49: moderate • 0.50 to 1.0: strong Significant or significant ordered difference across the groups
**Missing data**					
Descriptive statistics; frequencies and percentages of missing data (daily scores over time)	• MP • UF-DBD	• RND • EOT	AH	ASTEROID 1, patients with AH measurements: all patients except for Japanese centers^e^ and only US patients	
**Comparability of methods (AH, MP, UF-DBD)**					
1. Cross-tabulation of benchmark scores (HMB eligibility, responder status and amenorrhea, calculation of sensitivity, specificity, PPV and NPV) 2. Kaplan–Meier curves; descriptive statistics, histograms of difference	• MP • UF-DBD	• RND • EOT	AH	ASTEROID 1, patients with AH measurements	

*AH* Alkaline hematin method, *EOT* End of treatment, *ICC* Intraclass correlation coefficient, *MP (MP SAP-cv3)* Menstrual pictogram superabsorbent polymer-containing version 3, *NA* Not applicable/available, *NPV* Negative predictive value, *PPV* Positive predictive value, *PGI-S* Patient Global Impression of Severity, *PRO* Patient-reported outcomes, *RND* Randomization, *SF-36 v2®* Short-Form 36 Health Survey Version 2, *SCR* Screening, *T* Treatment, *UF-DBD* Uterine Fibroid Daily Bleeding Diary, *UF-DSD v3b* Uterine Fibroid Daily Symptom Diary version 3, *UF-IS v3* Uterine Fibroid Impact Scale version 3, *UFS-QoL* Uterine Fibroid Symptom and Quality of Life Questionnaire

^a^Monthly sum scores were based on the 28 days prior to and including the visit date; bleeding episode sum scores were collected on or closest to the visit date within 27 days prior

^b^ASTEROID 1 only

^c^For analyses including the AH method, only data from patients with AH measurements in ASTEROID 1 were used

^d^Use of total instrument scores only

^e^AH measurements were not performed in patients from Japanese centers; therefore, these patients were excluded from analysis

### Evaluation of psychometric and other measurement properties

This quantitative work aimed to evaluate the psychometric and other measurement properties of the newly developed PRO instruments with specific statistical analyses, including analyses of item performance/variability, test retest reliability, criterion, construct validity, and responsiveness (Table 1). In addition, missingness and the degree of comparability between the AH and both the MP and the UF-DBD were assessed as summarized in Table 1. Results presented are from ASTEROID 1 (where the AH method was used) and these are supported by data from ASTEROID 2, where indicated.

The MP is a PBAC used for the semi-quantitative evaluation of MBL. It comprises diagrams (icons) depicting a graded series of stained towels or tampons, and each icon is assigned a blood volume derived from measurements with the AH method. Patients respond to the MP whenever a sanitary product is changed, by choosing a pictogram icon and letter, based on the degree of staining of their sanitary products(s). Pictogram letters (a–f [towels] and a–d [tampons]) indicate staining intensity, with “a” the lowest intensity and “d” or “f” the highest intensity [15].

The UF-DBD is a single-item daily questionnaire, which assesses patient perceptions of vaginal bleeding severity on that day. Patients respond to the question ‘Rate the severity of any vaginal bleeding in the past 24 hours’ with “No vaginal bleeding,” “Spotting,” “Mild,” “Moderate,” “Severe,” or “Very severe”. The daily responses on the verbal rating scale were then assigned values of 0–10 (0=“No vaginal bleeding”, 1 = “Spotting”, 4 = “Mild”, 6 = “Moderate”, 8 = “Severe”, 10 = “Very Severe”), as informed by previous qualitative research involving the cognitive debriefing of the questionnaire in women with UF. Patient responses to the MP and the UF-DBD were collected on the same hand-held electronic device during ASTEROID 1 and 2 by the patients at home.

The other instruments referred to in this psychometric analysis have been described in the previous ASTEROID 1 and 2 publications [19, 20].

### Statistical methodology

Psychometric and other measurement properties of the MP and UF-DBD were analyzed using descriptive statistics including histograms, scatterplots, Spearman rank correlation coefficients and intra-class correlation coefficients, as well as Wilcoxon signed rank tests and the Jonckheere-Terpstra and Kruskal-Wallis tests. To this purpose, daily sums of mL blood loss from the MP and AH measurements were added up to sums of mL blood loss over 28 days (monthly scores) or over the bleeding episode (bleeding episode scores) preceding and including the respective visit at the clinical study site (e.g., randomization (RND), end of treatment (EOT) visit) in Asteroid 1 and 2. Similarly, the daily responses to the UF-DBD were aggregated over 28 days (monthly scores)/bleeding episodes (bleeding episode scores) and also aggregated scores for the reference measures UF-DSD v3 and UF-IS v3 (which were administered either daily or weekly, respectively) were derived as needed.

Test-retest reliability was assessed by the intraclass correlation coefficient (ICC) using Shrout-Fleiss reliability single score statistic among patients classified as stable between two consecutive timepoints, during screening and treatment phases, with the AH (ASTEROID 1) and the PGI-S (ASTEROID 1 and 2) used to define stable patients. In this respect, stable patients were defined as either a < 10 mL, < 20 mL, < 10%, < 20% difference in AH score or no change in the PGI-S score between the two assessments. There are no widely agreed benchmarks which can be used in the interpretation of the ICC. For the assessment of ICCs of the continuous MP, the thresholds proposed by Cicchetti (1994) [22] and Fleiss (1986) [23] for scores from continuous multi-item instruments were used: < 0.40 poor; 0.40–0.59 moderate; 0.60–0.74 good; > 0.75 excellent. Other thresholds exist, however, such as < 0.5 poor, 0.5–0.75 moderate, 0.75–0.9 good, > 0.90 excellent [24]. Since the ordinal UF-DBD is a single item instrument, lower ICCs for this measure were expected and a threshold of ≥0.50 was considered to indicate at least moderate reliability [24, 25].

For assessment of criterion and convergent/divergent validity, Spearman rank correlation coefficients were calculated. Using commonly accepted conventions, correlation coefficients values ranging from 0.10 to 0.29 were classed as weak correlations, from 0.30 to 0.49 as moderate correlations, and from 0.5 to 1.0 as strong correlations [26, 27]. For assessment of known groups validity, mean sum scores were compared for patient groups differing by AH-defined bleeding severity (3 groups based on MBL severity thresholds of 2 mL and 80 mL and 3 groups by AH tertile scores, each for monthly and bleeding episode sum scores) (ASTEROID 1) and severity of the condition assessed by the PGI-S (5 groups from “none” to “very severe”), for monthly sum scores only (ASTEROID 1 and 2). Responsiveness was evaluated using Spearman Rank correlation coefficients between change in the MP and the UF-DBD monthly and bleeding episode scores and the changes in reference measures from RND to EOT. Additionally, the difference in change scores in the MP and the UF-DBD between and within groups of patients classified by the degree of change using AH (ASTEROID 1) and the UF-DBD and the PGI-S (ASTEROID 1 and 2) was assessed.

Analyses of missingness and degree of comparability between the AH and both the MP, and the UF-DBD were conducted using descriptive statistics, cross-tabulation of relevant benchmark scores, Kaplan-Meier curves, and histograms of difference.

Further details of all statistical methods are included in Table 1.

Neither instrument alone could distinguish between data that was truly “missing” (i.e. no data record existed) and patients who had “no bleeding”. Therefore, MP and AH values were independently compared with UF-DBD entries as a means for distinguishing between the two conditions.

## Results

### Sanitary item and pictogram distribution

In ASTEROID 1 and 2, a total of 101,717 sanitary items were assessed; 75,500 towels and 26,217 tampons. Of these, 70,063 sanitary items were collected from ASTEROID 1, including 53,142 towels and 16,921 tampons. The proportion of sanitary products with an unspecific brand (termed “other”) was low, constituting 5.9% of all towels and 2.4% of all tampons in ASTEROID 1.

### Item performance/variability

In ASTEROID 1 and 2, all response options for the MP were used; MP response options for high staining intensity “e” and “d” were the most frequently reported by women during the 28 days prior to and including randomization, with 2498 (21.2%) reporting “e” and 2664 (63.9%) women reporting “d” using towels and tampons, respectively. During the last 4 weeks of treatment, including the EOT visit, low staining intensity was the most frequently reported response option in women using towels, with 556 (23.6%) reporting pictogram letter “a.” During the same time period, “d” was most frequently reported by 394 (62.2%) of women using tampons, indicating high staining intensity. Also, all response options of the UF-DBD were used.

### Psychometric analyses

The psychometric analysis of the MP and the UF-DBD with the AH method as reference was based on data from all patients from ASTEROID 1 (*N* = 623). Analysis, including AH data, was based on 528 patients, excluding 95 patients without AH measurements. Data from all patients in ASTEROID 2 (*N* = 228) were used for the supportive psychometric analysis of the MP and the UF-DBD as feasible.

### Test-retest reliability

In ASTEROID 1, the test-retest reliability ICC estimate (95% confidence interval [CI]) of the MP monthly scores was 0.93 (0.88–0.96) during screening (SCR2) and randomization periods and 0.96 (0.94–0.97) during treatment in AH-defined stable patients. The ICC estimate (95% CI) for the UF-DBD monthly scores was 0.88 (0.80–0.93) during screening and 0.88 (0.84–0.91) during treatment in AH-defined stable patients. Similarly, the ICC estimate was 0.86 (0.81–0.90) with MP monthly scores and 0.87 (0.82–0.91) with UF-DBD monthly scores during treatment in PGI-S-defined stable patients.

Observations in ASTEROID 2 using the PGI-S to define stable populations support the ASTEROID 1 results in general.

### Criterion validity

Strong correlations between the monthly MP and AH sum scores were observed at randomization (r_s_ = 0.72) and EOT (r_s_ = 0.97). Similarly, strong correlations were observed between the monthly UF-DBD and AH sum scores at EOT (r_s_ = 0.84) and moderate correlations at randomization (r_s_ = 0.44).

### Construct validity

#### Convergent and divergent validity

In ASTEROID 1, strong, positive correlations between the monthly MP and the UF-DBD sum scores were observed at randomization (r_s_ = 0.56) and EOT (r_s_ = 0.89), thus supporting convergent validity.

Weak correlations were hypothesized and observed between the MP monthly sum scores and the UF-DSD v3 (bloating/swelling and pain domain scores and total scores) at randomization and EOT (all r_s_ < 0.30). Furthermore, the MP demonstrated a largely weak-moderate correlation with other reference measures (UF-IS v3, UFS-QoL, and SF-36 v2®) at randomization and EOT (Table 2).

**Table 2:** Convergent and divergent validity analysis with MP and reference measures (monthly sum scores)

Spearman Rank Correlation Coefficient of MP monthly sum score to	RND		EOT	
	N	r_**s**_	N	r_**s**_
**UF-DBD** monthly sum score	283	0.56	263	0.89
**UF-DSD v3** monthly average bloating and swelling domain score	283	0.12	263	0.16
**UF-DSD v3** monthly average pain domain score	283	0.06	263	0.27
**UF-DSD v3** monthly average total score^a^	283	0.10	263	0.21
**UF-IS v3** monthly sum total score	263	0.21	247	0.35
**UFS-QoL** symptom domain score at visit	287	0.18	262	0.45
**UFS-QoL** HRQoL domain score at visit	282	−0.18	255	−0.41
**UFS-QoL** concerns related to soiling score at visit	282	0.20	257	0.51
**SF-36 v2®** physical component domain score at visit	278	−0.04	258	− 0.32
**SF-36 v2®** mental component domain score at visit	279	−0.12	258	−0.27
**SF-36 v2®** bodily pain subscale score at visit	286	−0.09	262	−0.31

Classification of Spearman rank correlation coefficient r_s_: Weak correlation: 0.10 < |r_s_| < 0.30, Moderate correlation: 0.30 ≤ |r_s_| < 0.50, Strong correlation: 0.50 ≤ |r_s_| < 1

All patients analyzed here had been enrolled in the ASTEROID 1 study with an existing date of visit

*EOT* End of treatment, *HRQoL* Health-related quality of life, *MP (MP SAP-c v3)* Menstrual pictogram superabsorbent polymer-containing version 3, *N* Number of patients, *RND* Randomization, *r*_*s*_ Spearman rank correlation coefficient, *SF-36 v2®* Short-Form 36 Health Survey Version 2, *UF-DBD* Uterine Fibroid Daily Bleeding Diary, *UF-DSD v3* Uterine Fibroid Daily Symptom Diary version 3, *UF-IS v3* Uterine Fibroid Impact Scale version 3, *UFS-QoL* Uterine Fibroid Symptom and Quality of Life Questionnaire

^a^UF-DSD v3 total score with exclusion of item 1 (UF-DBD)

Similarly, correlations of the monthly UF-DBD scores with reference measures other than the MP were weak at RND (all |r_s_| < 0.30) and largely moderate at EOT in general (Table 3). Correlations were moderate at EOT with the UFS-QoL domains (symptom, health-related quality of life, and concerns related to soiling).

**Table 3:** Convergent and divergent validity analysis with UF-DBD and reference measures (monthly sum scores)

Spearman Rank Correlation Coefficient of UF-DBD monthly sum score to	RND		EOT	
	N	r_**s**_	N	r_**s**_
**UF-DSD v3** monthly average total score^a^	286	0.19	266	0.29
**UF-DSD v3** monthly average bloating and swelling domain score	286	0.19	266	0.25
**UF-DSD v3** monthly average pain domain score	286	0.17	266	0.32
**UF-IS v3** monthly sum total score	262	0.24	250	0.38
**UFS-QoL** symptom domain score at visit	286	0.11	264	0.42
**UFS-QoL** HRQoL domain score at visit	281	−0.08	257	−0.36
**UFS-QoL** concerns related to soiling score at visit	281	0.12	259	0.46
**SF-36 v2®** physical component domain score at visit	277	−0.07	260	−0.29
**SF-36 v2®** mental component domain score at visit	278	−0.16	260	−0.24
**SF-36 v2®** bodily pain subscale score at visit	285	−0.19	264	−0.34

Classification of Spearman rank correlation coefficient r_s_: Weak correlation: 0.10 < |r_s_| < 0.30, Moderate correlation: 0.30 ≤ |r_s_| < 0.50, Strong correlation: 0.50 ≤ |r_s_| < 1

All patients analyzed here had been enrolled in the ASTEROID 1 study with an existing date of visit

*EOT* Indicates end of treatment, *HRQoL* Health-related quality of life, *N* Number of patients, *RND* Randomization, *r*_*s*_ Spearman rank correlation coefficient, *SF-36 v2®* Short-Form 36 Health Survey Version 2, *UF-DBD* Uterine Fibroid Daily Bleeding Diary, *UF-DSD v3* Uterine Fibroid Daily Symptom Diary version 3, *UF-IS v3* Uterine Fibroid Impact Scale version 3, *UFS-QoL* Uterine Fibroid Symptom and Quality of Life Questionnaire

^a^UF-DSD v3 total score with exclusion of item 1 (UF-DBD)

Overall, similar results from ASTEROID 2 (data not shown) confirm the convergent and divergent validity of the MP and the UF-DBD scores from ASTEROID 1 data.

#### Known-groups validity

Mean monthly and bleeding episode MP and UF-DBD sum scores increased in monotonic fashion with greater AH-defined and PGI-S-defined disease severity at randomization and EOT (Tables 4 and 5). The differences between the disease severity groups were substantial or statistically significant (*P* < 0.05).

**Table 4:** Known groups validity analysis with MP and reference measures (monthly sum scores)

Time period	Groups	N	Mean (SD)	Test statistic/***P*** value^**a**^	Test statistic/***P*** value^**b**^
**Differences in MP SAP-c v3 monthly sum scores between groups defined by AH method (MBL severity thresholds of 2 mL and 80 mL defined by clinical rationale)**					
**RND**	Group 1 (AH monthly sum score < 2 mL)	12	85.84 (102.89)	7374.0 / <.0001	46.5 / <.0001
	Group 2 (2 ≤ AH monthly sum score < 80 mL)	42	137.46 (142.08)		
	Group 3 (AH monthly sum score ≥ 80 mL)	161	224.92 (130.08)		
**EOT**	Group 1 (AH monthly sum score < 2 mL)	144	0.72 (6.38)	8078.0 / <.0001	167.0 / <.0001
	Group 2 (2 ≤ AH monthly sum score < 80 mL)	25	73.24 (45.53)		
	Group 3 (AH monthly sum score ≥ 80 mL)	27	210.19 (158.00)		
**Known groups validity: Differences in MP monthly sum scores between groups defined by AH method (thresholds defined by AH tertiles)**					
**RND**	Group 1 (AH monthly sum score Tertile 1)	72	129.19 (118.69)	12,791.0 / <.0001	98.6 / <.0001
	Group 2 (AH monthly sum score Tertile 2)	72	173.04 (72.45)		
	Group 3 (AH monthly sum score Tertile 3)	71	299.36 (150.48)		
**EOT**	Group 1/2^c^ (AH monthly sum score Tertile 1 + Tertile 2)	136	0.61 (6.53)	8127.0 / <.0001	177.8 / <.0001
	Group 3 (AH monthly sum score Tertile 3)	60	125.44 (135.40)		
**Differences in MP monthly sum scores between groups defined by PGI-S**					
**RND**	Group 1 (PGI-S = 1,2 [None/Very mild])	15	159.40 (94.96)	17,216.5 / <.0001	19.2 / 0.0007
	Group 2 (PGI-S = 3 [Mild])	24	169.23 (67.59)		
	Group 3 (PGI-S = 4 [Moderate])	92	200.22 (130.95)		
	Group 4 (PGI-S = 5 [Severe])	98	202.27 (136.39)		
	Group 5 (PGI-S = 6 [Very severe])	52	287.05 (199.23)		
**EOT**	Group 1 (PGI-S = 1,2 [None/Very mild])	98	12.80 (45.40)	15,628.0 / <.0001	30.5 / <.0001
	Group 2 (PGI-S = 3 [Mild])	56	28.30 (54.07)		
	Group 3 (PGI-S = 4 [Moderate])	62	54.98 (97.59)		
	Group 4 (PGI-S = 5 [Severe])	35	116.31 (186.47)		
	Group 5 (PGI-S = 6 [Very severe])	9	54.13 (122.29)		

“All patients with AH measurements from ASTEROID 1” refers to all patients enrolled with an existing date of visit and AH or PGI-S measurements

RND Tertile 1: (0.00 to 101.24), Tertile 2: (> 101.24 to 203.73), Tertile 3: (> 203.73 to 1164.18)

EOT Tertile 1 and Tertile 2: (0.00 to 0.00), Tertile 3: (> 0.00 to 747.94)

*AH* Alkaline hematin method, *EOT* End of treatment, *MBL* Menstrual blood loss, *MP (MP SAP-c v3)* Menstrual pictogram superabsorbent polymer-containing version 3, *N* Number of patients, *PGI-S* Patient Global Impression of Severity, *RND* Randomization, *SD* Standard deviation

^a^Jonckheere-Terpstra test

^b^Kruskal-Wallis test

^c^As the first and the second tertile are the same at EOT, only two groups based on tertiles were defined

**Table 5:** Known groups validity analysis with UF-DBD and reference measures (monthly sum scores)

Time period	Groups	N	Mean (SD)	Test statistic/***P*** value^**a**^	Test statistic/***P*** value^**b**^
**Differences in UF-DBD monthly sum scores between groups defined by AH method (MBL severity thresholds of 2 mL and 80 mL defined by clinical rationale)**					
**RND**	Group 1 (AH monthly sum score < 2 mL)	12	20.33 (14.37)	6464.0 / <.0001	21.7 / <.0001
	Group 2 (2 ≤ AH monthly sum score < 80 mL)	42	27.31 (11.50)		
	Group 3 (AH monthly sum score ≥ 80 mL)	161	36.10 (15.95)		
**EOT**	Group 1 (AH monthly sum score < 2 mL)	144	1.31 (3.81)	7847.5 / <.0001	133.5 / <.0001
	Group 2 (2 ≤ AH monthly sum score < 80 mL)	25	24.24 (19.75)		
	Group 3 (AH monthly sum score ≥ 80 mL)	27	33.11 (17.95)		
**Differences in UF-DBD monthly sum scores between groups defined by AH method (thresholds defined by AH tertiles)**					
**RND**	Group 1 (AH monthly sum score Tertile 1)	72	26.93 (13.15)	10,791.0 / <.0001	37.1 / <.0001
	Group 2 (AH monthly sum score Tertile 2)	73	31.78 (9.85)		
	Group 3 (AH monthly sum score Tertile 3)	70	42.06 (19.21)		
**EOT**	Group 1/2^c^ (AH monthly sum score Tertile 1 + Tertile 2)	136	1.20 (3.83)	7872.5 / <.0001	132.0 / <.0001
	Group 3 (AH monthly sum score Tertile 3)	60	25.42 (19.92)		
**Differences in UF-DBD monthly sum scores between groups defined by PGI-S**					
**RND**	Group 1 (PGI-S = 1,2 [None/Very mild])	15	28.00 (9.97)	16,584.0 / 0.0011	12.0 / 0.0171
	Group 2 (PGI-S = 3 [Mild])	24	30.50 (7.68)		
	Group 3 (PGI-S = 4 [Moderate])	90	33.71 (13.53)		
	Group 4 (PGI-S = 5 [Severe])	97	33.61 (13.84)		
	Group 5 (PGI-S = 6 [Very severe])	54	42.24 (21.02)		
**EOT**	Group 1 (PGI-S = 1,2 [None/Very mild])	99	4.18 (9.04)	15,835.5 / <.0001	28.8 / <.0001
	Group 2 (PGI-S = 3 [Mild])	56	8.82 (15.19)		
	Group 3 (PGI-S = 4 [Moderate])	62	10.02 (14.16)		
	Group 4 (PGI-S = 5 [Severe])	36	24.22 (29.52)		
	Group 5 (PGI-S = 6 [Very severe])	9	10.89 (20.51)		

“All patients with AH measurements from ASTEROID 1” refers to all patients enrolled with an existing date of visit and AH or PGI-S measurements

RND Tertile 1: (0.00 to 101.24), Tertile 2: (> 101.24 to 203.73), Tertile 3: (> 203.73 to 1164.18)

EOT Tertile 1 and Tertile 2: (0.00 to 0.00), Tertile 3: (> 0.00 to 747.94)

*AH* Alkaline hematin method, *EOT* End of treatment, *MBL* Menstrual blood loss, *N* Number of patients, *PGI-S* Patient Global Impression of Severity, *RND* Randomization, *SD* Standard deviation, *UF-DBD* Uterine Fibroid Daily Bleeding Diary

^a^Jonckheere-Terpstra test

^b^Kruskal-Wallis test

^c^As the first and the second tertile are the same at EOT, only two groups based on tertiles are defined

Observations in ASTEROID 2 were similar for both the MP and UF-DBD for patients grouped according to PGI-S severity only (data not shown).

### Responsiveness

As a priori hypothesized, strong association was observed between changes in MP monthly sum scores and UF-DBD monthly sum scores (r_s_ = 0.75) or changes in AH monthly sum scores (r_s_ = 0.86) from randomization to EOT. Moderate to weak associations between change scores were found with the UF-IS v3 monthly sum scores (r_s_ = 0.35), the UFS-QoL (|r_s_| < 0.40), the UF-DSD v3 (r_s_ < 0.25) and the PGI-S (r_s_ = 0.21), from randomization to EOT. As expected, differences in the changes in MP monthly sum scores between groups defined by AH changes (*P* < 0.0001) and PGI-S change (improvement/no change/deterioration, *P* = 0.0251; and *P* = 0.0040 ordered differences) were large and statistically significant. Also, large and statistically significant differences between mean changes in monthly UF-DBD sum scores between groups defined by AH change categories (*P* < 0.0001) and PGI-S (*P* < 0.01) were identified.

Observations in ASTEROID 2 were similar for both the MP and UF-DBD for strengths of associations between changes in the MP or the UF-DBD and the reference measures from randomization to EOT and MP and UF-DBD score changes grouped according to PGI-S severity only (data not shown).

### Analyses of missingness

Overall in ASTEROID 1, more sanitary protection items were evaluated by the MP compared with the AH method; 44,230 MP and 39,855 AH items. Of the total 241 patients in the screening period, the mean absolute (mean relative) frequency of missing data was 2.8 (9.9%) days for MP values, 2.8 (9.9%) days for UF-DBD entries, and 3.1 (11.2%) days for AH measurements (Table 6). Of all 223 patients during the 28 days prior to EOT, the mean absolute (mean relative) frequency of missing data was 3.0 (11.1%) days for MP values, 2.9 (10.6%) days for UF-DBD entries, and 3.2 (11.6%) days for AH measurements.

**Table 6:** Absolute frequency of missing daily AH, MP values, and daily UF-DBD scores per patient

Patient group	Number of patients	Average number of days in period	Mean	SD	Median	IQR (Q1-Q3)	Min-Max
***Screening period***							
*Patients with AH measurements*							
Daily AH values	241	28	3.1	3.7	2.0	0.0–5.0	0.0–21.0
Daily MP values	241	28	2.8	3.6	1.0	0.0–4.0	0.0–19.0
Daily UF-DBD scores	241	28	2.8	3.5	1.0	0.0–4.0	0.0–21.0
*US Patients with AH measurements*							
Daily AH values	92	28	4.6	4.3	3.5	1.0–6.5	0.0–21.0
Daily MP values	92	28	4.1	4.2	3.0	1.0–6.0	0.0–19.0
Daily UF-DBD scores	92	28	4.3	4.2	3.5	1.0–6.0	0.0–21.0
***28 days prior to and including EOT***							
*Patients with AH measurements*							
Daily AH values	223	28	3.2	4.1	2.0	0.0–4.0	0.0–21.0
Daily MP values	223	28	3.0	4.0	2.0	0.0–4.0	0.0–21.0
Daily UF-DBD scores	223	28	2.9	3.8	2.0	0.0–4.0	0.0–21.0
*US Patients with AH measurements*							
Daily AH values	82	28	5.0	4.9	4.0	2.0–7.0	0.0–21.0
Daily MP values	82	28	4.8	4.7	4.0	1.0–7.0	0.0–21.0
Daily UF-DBD scores	82	28	4.6	4.4	4.0	1.0–6.0	0.0–21.0

The patient group analyzed here refers to those who had AH measurements from any study center. Patients from Japan were excluded as they were not asked to collect sanitary items for the AH method

*AH* Alkaline hematin method, *EOT* End of treatment, *IQR* Interquartile range, *Max* Maximum, *Min* Minimum, *MP (MP SAP-c v3)* Menstrual pictogram superabsorbent polymer-containing version 3, *N* Number of patients, *UF-DBD* Uterine Fibroid Daily Bleeding Diary

### Comparability of methods

#### Patient eligibility

HMB (MBL > 80 mL) was identified in 59.0% (181/307) and 75.2% (231/307) of women during the screening period using the AH method and MP, respectively. For assessing the presence of HMB, compared with the AH method, the MP presented a sensitivity of 96.7% (175/181) and specificity of 55.6% (70/126) in assessing HMB, and the positive predictive value (PPV) was 75.8% (175/231).

#### Amenorrhea

In ASTEROID 1, amenorrhea (MBL < 2 mL) was detected in 73.5% (144/196) and 70.4% (138/196) of women (with AH measurements and data to assess amenorrhea) using the AH method and MP, respectively. For assessment of amenorrhea, the MP demonstrated a sensitivity of 95.8% (138/144) and specificity of 100.0% (52/52). In addition, the UF-DBD was 93.8% sensitive and 98.1% specific in the detection of amenorrhea.

#### Heavy menstrual bleeding response

In ASTEROID 1, HMB response (MBL < 80 mL and > 50% reduction) during treatment, compared with baseline, was indicated in 76.2% (138/181) and 75.7% (137/181) of women (with data to assess HMB response) using the AH method and the MP, respectively. The PPV of the MP was 99.3%.

#### Time to onset of amenorrhea

Time to onset of amenorrhea was calculated with a mean (standard deviation) difference of − 0.6 (6.3) days and the time to onset of controlled bleeding difference was − 1.8 (10.7) days when the events were assessed using the AH method, compared with the MP. The onset of controlled bleeding and amenorrhea was detected slightly later with the MP, compared with the AH method, although the time course of the overall Kaplan-Meier curves for these two instruments appeared similar (data not shown).

## Discussion

A large proportion of women clinically diagnosed with UF experience HMB, and severe cases of HMB can have a considerable impact on different aspects of women’s’ lives [9, 10, 28] and limit participation in daily activities [9]. The development of the semi-quantitative MP for assessment of MBL suitable for use with modern sanitary protection as well as the UF-DBD for assessment of subjective bleeding severity can facilitate both clinical research and practice.

In ASTEROID 1 and 2, in general, the full range of MP and UF-DBD response options were used. The score distributions reflect the cyclic character of the disease with symptoms being concentrated, but not limited to, the time of bleeding (typically to 5–10 days per month).

Overall, the psychometric and measurement properties of the MP and the UF-DBD were found to be appropriate.

The test-retest reliability of the MP and the UF-DBD was excellent and acceptable, respectively, in stable patients (defined by both the AH method and the PGI-S). However, this finding must be considered with caution, due to the potential limitations in establishing test-retest reliability in relapsing/episodic diseases (such as menstrual bleeding), as highlighted by the FDA [21].

Strong correlations observed between monthly MP and AH sum scores for MBL confirm the criterion validity of the MP. This was also shown for the UF-DBD, which exhibited a lower correlation with the AH sum scores; these results, however, were expected a priori. The UF-DBD assesses women’s perception of vaginal bleeding severity. This may include aspects beyond pure quantity and thus correlations with actual quantity of blood volume may not be as strong as correlations observed between measures of bleeding volume only.

Construct validity of both the MP and the UF-DBD was determined via establishment of convergent, divergent and known-groups validity. As expected, correlations of the MP monthly and bleeding episode scores with the UF-DBD scores were strong, supporting the convergent validity of both bleeding assessment instruments.

Correlations of both the MP and the UF-DBD with the other instruments (UF-DSD v3 and UF-IS v3) were weak to moderate in nature, as a priori hypothesized, likely due to the differences in the different concepts and disease aspects covered by each of these instruments.

In general, the mean monthly MP and UF-DBD scores increased with higher AH scores and bleeding volume and PGI-S severity, with substantial and significant differences seen between the known severity groups. Pronounced group differences in MP and UF-DBD sum scores well reflected the known-groups categories defined by the AH method and PGI-S.

Responsiveness was supported by strong associations between changes in the monthly sum scores of MP and UF-DBD respectively with those in AH, and those between changes in the MP and in the UF-DBD. Furthermore, there were large and significant differences in change in the MP and UF-DBD monthly sum scores between the groups defined by AH and PGI-S change categories.

The psychometric findings from the ASTEROID 2 largely confirm those from the ASTEROID 1 analyses; however, as the AH method was not applied in ASTEROID 2 analyses requiring the AH method as a reference measure could not be conducted. Of note, approximately 70,000 sanitary products were used in ASTEROID 1, with a higher number of sanitary products rated by the MP than with the AH method. The small percentage of unknown brands of sanitary products reported in the study supports the compliance with the study protocol and the reliability of the collected data, as unknown brands of sanitary products may not contain SAP granules, which could affect their absorbance and staining characteristics.

Missingness analysis demonstrates that evaluations were more frequently made by the MP than the AH method, with the AH method thus associated with a higher frequency of missing data. Furthermore, analyses indicated the mean absolute (relative) frequency of days with missing values per patient, and of missing daily values per patient, was higher with the AH method than with the MP or the UF-DBD.

Comparability analysis of the MP, UF-DBD, and AH methods to identify treatment eligibility indicated that approximately 24% of women selected by the MP would not have been classed as eligible for study participation if assessed for HMB by the AH method. It is important to note, however, that women with an MBL volume slightly less than 80 mL as rated by the AH method may still suffer from a similar severity perception of the disease, compared with those with “HMB” of 80 ml or slightly above rating by the AH method. Regarding treatment response, the MP and the UF-DBD both offered greater than 90% sensitivity in detecting amenorrhea, and the MP was almost 99% sensitive in detecting HMB response.

There are several limitations to this study. First, analysis on psychometric and other measurement properties were conducted post hoc using datasets from ASTEROID 1 and 2 studies intended for assessment of efficacy and safety of the novel selective progesterone receptor modulator vilaprisan.

Therefore, although for the assessment of psychometric and other measurement properties the overall data handling process was in line with that employed in the ASTEROID 1 and 2 studies, some deviations were necessary including applying scoring for UF-DBD, score aggregation, biopsy-related handling of bleeding data, and limited missing data imputation. Therefore, deviations between the results presented and the ASTEROID 1 and 2 clinical efficacy and safety study results may exist. Bleeding episode data from the two interventional studies was also difficult to interpret due to the high number of patients during the study conduct without bleeding and with consequently undefined bleeding episodes as a consequence of positive treatment effect. In addition, determination of matching time points for assessment between the MP and the UF-DBD and the reference measures was difficult due to the post hoc study design. Finally, the AH method, as the most important reference measure, was not employed in ASTEROID 2; therefore, only limited data to confirm the analysis of ASTEROID 1 data were available. Therefore, these analyses should be replicated with MP and AH data collected from other clinical studies.

## Conclusions

Overall, the analyses presented here demonstrate favorable psychometric and other measurement properties of the MP and UF-DBD. These instruments were associated with a lower frequency of missing patient data compared to the AH method. In general, however, a good agreement with the standard AH method could be shown. The results support the use of the MP and the UF-DBD to assess clinical efficacy endpoints in UF phase III studies, replacing the AH method.

## Data Availability

The datasets used and/or analysed during the current study are available from the corresponding author on reasonable request.

## Abbreviations

AH: Alkaline hematin
CI: Confidence interval
EOT: End of treatment
FDA: US Food and Drug Administration
GCP: Good Clinical Practice
HMB: Heavy menstrual bleeding
HRQoL: Health-related quality of life
ICC: Intra-class correlation coefficient
MBL: Menstrual blood loss
MP SAP-c-v3: Menstrual pictogram superabsorbent polymer-containing version 3
NA: Not applicable/available
NPV: Negative predictive value
PBAC: Pictorial blood loss assessment charts
PGI-S: Patient Global Impression of Severity
PPV: Positive predictive value
PRO: Patient-reported outcomes
RND: Randomization
SAP: Superabsorbent polymer
SF-36 v2®: Short-Form 36 Health Survey Version 2
T: Treatment
UF: Uterine fibroids
UF-DBD: Uterine Fibroid Daily Bleeding Diary
UF-DSD v3: Uterine Fibroid Daily Symptom Diary version 3
UF-IS v3: Uterine Fibroid Impact Scale version 3
UFS QoL: Uterine Fibroid Symptom and Quality of Life Questionnaire
